# Threat imminence reveals links among unfolding of anticipatory physiological response, cortical-subcortical intrinsic functional connectivity, and anxiety

**DOI:** 10.1016/j.ynstr.2022.100428

**Published:** 2022-01-04

**Authors:** Rany Abend, Sonia G. Ruiz, Mira A. Bajaj, Anita Harrewijn, Julia O. Linke, Lauren Y. Atlas, Anderson M. Winkler, Daniel S. Pine

**Affiliations:** a Emotion and Development Branch, National Institute of Mental Health, National Institutes of Health, Bethesda, MD, 20892, USA; b Department of Psychology, Yale University, New Haven, CT, 06511, USA; c Department of Psychology, Education and Child Studies, Erasmus University Rotterdam, Rotterdam, the Netherlands; d National Center for Complementary and Integrative Health, National Institutes of Health, Bethesda, MD, 20892, USA

**Keywords:** Anxiety, Fear, Threat, Anticipation, Amygdala, Cortex

## Abstract

Excessive expression of fear responses in anticipation of threat occurs in anxiety, but understanding of underlying pathophysiological mechanisms is limited. Animal research indicates that threat-anticipatory defensive responses are dynamically organized by threat imminence and rely on conserved circuitry. Insight from basic neuroscience research in animals on threat imminence could guide mechanistic research in humans mapping abnormal function in this circuitry to aberrant defensive responses in pathological anxiety.

50 pediatric anxiety patients and healthy-comparisons (33 females) completed an instructed threat-anticipation task whereby cues signaled delivery of painful (threat) or non-painful (safety) thermal stimulation. Temporal changes in skin-conductance indexed anxiety effects on anticipatory responding as function of threat imminence. Multivariate network analyses of resting-state functional connectivity data from a subsample were used to identify intrinsic-function correlates of anticipatory-response dynamics, within a specific, distributed network derived from translational research on defensive responding.

By considering threat imminence, analyses revealed specific anxiety effects. Importantly, pathological anxiety was associated with excessive deployment of anticipatory physiological response as threat, but not safety, outcomes became more imminent. Magnitude of increase in threat-anticipatory physiological responses corresponded with magnitude of intrinsic connectivity within a cortical-subcortical circuit. Moreover, more severe anxiety was associated with stronger associations between anticipatory physiological responding and connectivity that ventromedial prefrontal cortex showed with hippocampus and basolateral amygdala, regions implicated in animal models of anxiety.

These findings link basic and clinical research, highlighting variations in intrinsic function in conserved defensive circuitry as a potential pathophysiological mechanism in anxiety.

## Highlights

• We translate animal insight on fear dynamics into human research on anxiety.
• Patients and controls completed threat anticipation task and resting-state scan.
• We observed aberrant threat-anticipatory physiological response dynamics in anxiety.
• Aberrant dynamics in anxiety linked to cortical-subcortical intrinsic function.
• Findings link basic, clinical research; highlight potential pathophysiological mechanism.

## 1 Introduction

Defensive responses in anticipation of threat are adaptive, but their excessive expression is a hallmark characteristic of pathological anxiety (Adolphs, 2013; Fanselow et al., 1988; Hamm, 2019; LeDoux, 2000; Rosen and Schulkin, 1998; Corchs and Schiller, 2019). The conserved nature of defensive responses can guide research on their aberrant expression in anxiety. While translational research indicates that defensive behaviors unfold with threat *imminence,* or proximity, studies to date have yet to consider threat imminence in examining aberrant threat-anticipation processes in anxiety and their underlying pathophysiological mechanisms. Here, we begin to address this gap.

Animal research indicates that defensive behaviors are dynamically organized by *threat imminence* and available behavioral options (Adolphs, 2013; Fanselow et al., 1988; Mobbs et al., 2020; LeDoux and Daw, 2018; Mobbs, 2018; Fox and Shackman, 2019). Thus, when a threat appears (a phase typically referred to as *encounter*/*post-encounter*), animals display freezing or passive avoidance behavior to evade detection and assess risks; as the threat looms closer and attack is imminently anticipated (*circa-strike* phase), acute defensive behaviors, such as active avoidance, become prominent (Fanselow et al., 1988; Rosen and Schulkin, 1998; Mobbs, 2018; Blanchard and Blanchard, 1989; Choi and Kim, 2010). Distinct patterns of physiological responding, including changes in heart rate and skin conductance, accompany the unfolding of these adaptive defensive behaviors, promoting their execution (Hamm, 2019; Rosen and Schulkin, 1998; Naqvi et al., 2006; Ojala and Bach, 2020). Recent research begins to extend such findings to humans (Hamm, 2019; Mobbs, 2018; Roelofs, 2017; Mobbs et al., 2009; Low et al., 2008; Qi et al., 2018; Wendt et al., 2017; Terburg et al., 2018).

Although threat-anticipatory responses are inherently adaptive, their excessive expression is central in the presentation of pathological anxiety (American Psychiatric Asso, 2013; Abend et al., 2020a), among the most prevalent psychiatric conditions (Kessler et al., 2005). From a translational-evolutionary perspective, anxiety could arise from aberrant function in the circuitry generating defensive responses, leading to maladaptive expression (Rosen and Schulkin, 1998; Corchs and Schiller, 2019; Mobbs, 2018; Robinson et al., 2019; Grupe and Nitschke, 2013). Prior work on anxiety primarily examines aggregated indices of physiological responses (average/maximal response across an anticipation window) in the context of potential threat, providing important clinical insight (Rosen and Schulkin, 1998; Robinson et al., 2019; Lissek et al., 2005). However, such analyses do not leverage translational insight on threat imminence as an organizing principle of defensive responding. Linking anxiety to aberrant *dynamics* of defensive responding could inform translational research on pathophysiology (Adolphs, 2013; Mobbs, 2018; Abend et al., 2020a; Grupe and Nitschke, 2013; Barlow, 2002). Our first aim examines whether threat imminence reveals novel associations between temporal patterns of defensive responding and anxiety.

Animal research consistently links defensive responses to a specific, distributed network of brain structures that includes amygdala (proper and extended) nuclei, hippocampus, hypothalamus, midbrain, and others, as well as specific portions of prefrontal cortex (Adolphs, 2013; Fanselow et al., 1988; Mobbs, 2018; Fox and Shackman, 2019; Terburg et al., 2018; McNaughton and Corr, 2004). Recent work begins to translate such findings to human-subject paradigms (Hamm, 2019; Mobbs, 2018; Roelofs, 2017; Mobbs et al., 2009; Low et al., 2008; Qi et al., 2018; Wendt et al., 2017; Terburg et al., 2018; McMenamin et al., 2014; Hur et al., 2020), but characterization of functional links within this circuitry is still limited. Importantly, no work directly links variation in circuitry function to aberrant expression of threat-anticipatory responses in anxiety. Given cross-species findings, we hypothesize that excessive threat-anticipatory responding in anxiety relates to function within this “defensive response” circuitry, including cortical-subcortical connections that drive the selection, maintenance, and regulation of imminence-specific defensive responses (Mobbs, 2018; Robinson et al., 2019; Hagihara et al., 2021; Myers-Schulz and Koenigs, 2012; Halladay and Blair, 2015).

Linking variations in the *intrinsic* function of this circuitry and aberrant expression of threat-anticipatory responses could potentially help identify individuals prone to anxiety (American Psychiatric Asso, 2013; Abend et al., 2020a). Resting-state functional connectivity (rsFC), indexing correlated neural activity fluctuations across functionally-related regions, could be used to identify intrinsic variations in circuitry function. rsFC data are increasingly used to characterize the organization of distributed networks (Biswal et al., 1997; Greicius et al., 2003; van den Heuvel and Hulshoff Pol, 2010) with high reliability and sensitivity to inter-subject differences (Finn et al., 2015; Van Dijk et al., 2010; Salvo et al., 2021). Growing evidence suggests that psychopathology manifests in aberrant rsFC patterns, potentially indicative of intrinsic perturbations or biases in specific functional networks (Northoff, 2015, 2020; Martino et al., 2016; Spellman and Liston, 2020; Xu et al., 2019; Canario et al., 2021; Viviano et al., 2018; Whitfield-Gabrieli et al., 2020). Such biases may thus serve as potential markers and treatment targets for pathological neurobiological processes (Whitfield-Gabrieli et al., 2020; Hohenfeld et al., 2018; Siddiqi et al., 2020; Parkes et al., 2020; Salomons et al., 2014; FitzGerald, 2016). However, it is imperative to link functional variations in specific circuits to aberrant behavior related to these circuits in order to make such inferences. Our second aim is to link intrinsic-function variations in the defensive response network and imminence-dependent defensive responding, as these relate to anxiety severity.

Here, we induced threat anticipation in a sample of healthy and clinically-anxious youth using cues instructed to signal subsequent delivery of highly-painful (threat) or non-painful (safety) thermal stimulation. We indexed the unfolding of anticipatory responding with threat imminence by measuring temporal changes in skin conductance (Hamm, 2019; Low et al., 2008; Wendt et al., 2017; Critchley et al., 2011). Drawing from animal research findings, we focused on two imminence-dependent effects that could manifest in anxiety (Blanchard and Blanchard, 1989; Choi and Kim, 2010): initial response to cue onset (corresponding to threat *encounter* phase) and increase in response with increasing threat imminence (*circa-strike*). We hypothesized that more severe anxiety is associated with greater physiological responses to both (American Psychiatric Asso, 2013). Network analyses on rsFC data from a different visit were then used to link anticipatory responding to variation in the intrinsic, multivariate functional organization of the defensive response circuitry. We hypothesized that connectivity within the circuitry correlates with magnitude of responding. Further, we hypothesized that anxiety severity covaries with the magnitude of associations between cortical-subcortical function and threat-anticipatory responding, indicative of intrinsic tendency for abnormal threat responding (Myers-Schulz and Koenigs, 2012; Halladay and Blair, 2015; Phillips et al., 2008; Lefler et al., 2020; Schiller and Delgado, 2010).

## 2 Material and methods

**Participants**. Data were from a sample of 50 youths recruited to participate in research on fear and anxiety at the National Institute of Mental Health (NIMH). This sample included 25 medication-free, treatment-seeking youth with pediatric anxiety disorders (17 females; *M*_age_ = 14.06 years, range = 9.34–17.90) and 25 healthy comparisons (HC; 16 females; *M*_age_ = 14.90 years, range = 9.30–17.28). See supplement for inclusion/exclusion criteria. Of note, we studied youth since anxiety typically emerges in early age and precedes additional, compounding psychopathology (Kessler et al., 2005; Kessler and Wang, 2008; Beesdo et al., 2009); studying youth with anxiety and no other disorders enables us to tightly link observed effects specifically to anxiety. Groups did not differ in sex, mean age, or mean IQ, *p*s > 0.17; as expected, the anxiety group reported markedly higher mean current anxiety symptoms (see below), *t*(30.44) = 11.87, *p* < 0.001. Written informed consent was obtained from parents of participants; written assent was obtained from youth. Procedures were approved by the NIMH Institutional Review Board. Participants received monetary compensation. Patients completed the study prior to treatment.

All 50 participants completed a threat anticipation task in the lab (outside the scanner) and their data were used in analysis of task-derived physiological data. Raw physiological data have been analyzed in a previous report using different analytic methods, in which we noted greater averaged overall anticipatory physiological responding in patients (Abend et al., 2021). Importantly, we did not previously consider the chronometry of physiological responses, nor any functional imaging, which are the focus of this report. For the current report, a subsample of 37 participants provided resting-state functional imaging data in a different visit. Thus, all physiology indices and analyses reported here, as well as all functional imaging data, have not been used before.

**Anxiety severity**. All participants were carefully assessed by trained clinicians using a structured clinical interview (Kaufman et al., 1997). The anxiety group was composed of treatment-seeking, medication-free youth who received a diagnosis of anxiety disorder and no other psychiatric disorders; the HC group was composed of youth without any psychiatric disorders. Analyses also considered anxiety severity dimensionally across the sample using the parent-reported Screen for Child Anxiety Related Emotional Disorders (SCARED)(Birmaher et al., 1997), to examine dimensional mechanistic variations. This “gold standard” measure of anxiety symptom severity includes 41 items pertaining to anxiety-related symptoms or behaviors; each item is rated on a 3-point Likert-type scale (0 = not true, 2 = very true); the total score was used in analyses. SCARED data were collected in each visit and averaged to improve consistency. Mean (SD) total score was 6.39 (3.21) in the HC group and 28.48 (10.68) in the anxiety group. See Fig. S1 for distribution of scores.

**Fig. 1:**
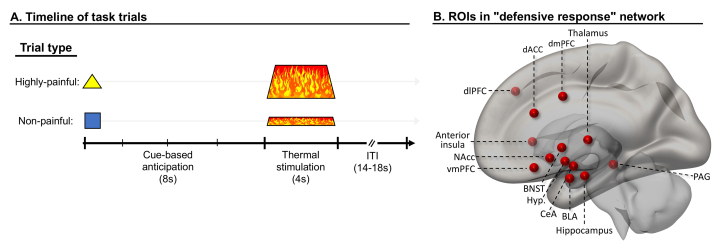
**Threat-anticipation task and defensive response network ROIs**. A) Timeline of task trials. The task was administered outside the scanner. Each trial started with presentation of cue instructed to predict highly-painful or non-painful thermal stimulation (temperatures individually calibrated before task), initiating an anticipation phase (8s). Next, thermal stimulation was applied (4s), followed by an intertrial interval (ITI; 14–18s) which included pain ratings. Analyses focused on anticipation phase. B) Literature-guided network of ROIs (regions of interest; here, showing just one hemisphere, in a mid-sagittal view with transparent cortex) selected for targeted analyses linking intrinsic functional connectivity and threat-anticipatory physiological responding. ROIs presented as spheres for display purposes only; ROIs in analyses were defined according to anatomical/functional boundaries using expert segmentations (see Methods and supplement). *Note*: dlPFC = dorsolateral prefrontal cortex, dACC = dorsal anterior cingulate cortex, vmPFC = ventromedial prefrontal cortex, dmPFC = dorsomedial prefrontal cortex, BNST = bed nucleus of the stria terminalis, Hyp. = hypothalamus, CeA = central nucleus of the amygdala, BLA = basolateral amygdala, NAcc = nucleus accumbens, PAG = periaqueductal gray.

**Threat-anticipation task.** Participants completed a threat anticipation task in the lab (outside the scanner) described in detail in previous work (Abend et al., 2021; Michalska et al., 2018; Atlas et al., 2010); see Fig. 1A. Briefly, shape cues were paired with individually-calibrated thermal stimulation applied to the forearm. Participants were instructed that one shape predicted non-painful stimulation (safety) while the other predicted highly painful stimulation (threat). We chose to use noxious thermal stimulation as it is a primary, robust reinforcer signaling potential physical damage. In each trial, participants were presented with the safety or threat cue (2s). Eight seconds after cue onset, participants received the relevant painful or non-painful thermal stimulation (4s, plus 0.5s ramp up and down). After variable delay (5–7s), participants provided pain ratings using a mouse (0–10 scale). Variable inter-trial interval (4–6s) separated trials. Eighteen trials featuring each of the two cues, in counterbalanced order, were administered. See supplement for additional details and data collected in the original task.

**Psychophysiological recording and processing.** Skin conductance level (SCL) data were used to assess anticipatory physiological responding (Hamm, 2019; Critchley et al., 2011), recorded continuously at 1000Hz from the left middle and index fingers using Biopac equipment and AcqKnowledge software (Goleta, CA). Following prior work (Abend et al., 2016, 2021), continuous deconvolution (*Ledalab* MATLAB package) was used to decompose SCL data into tonic (slow-changing) and phasic (fast-changing impulse response) components (Benedek and Kaernbach, 2010).

**Physiological data analysis**. The combination of instructed contingencies and fixed anticipation window enabled assessment of precise temporal effects in the unfolding of anticipatory defensive responding. Accordingly, analyses focused on changes in physiological response within the 8-s, cue-based anticipation phase starting with cue onset and ending with thermal stimulation delivery. Outcome imminence was operationalized as time between cue onset and outcome delivery.

Within the anticipation window, SCL data were assessed in four 2-s, non-overlapping, successive bins. Within each bin, we derived the average SCL signal and subtracted from it the mean tonic SCL level for that bin; this served as the index of physiological response in analyses. This index enabled us to assess the magnitude of overall physiological responding in each bin while controlling for individual differences in local tonic signal and responsivity, and without making assumptions on response functions or relying on pre-cue epochs to derive a baseline. That is, skin conductance analyses in this context typically focus on skin conductance responses that either assume discrete, canonical responses or index maximal response in relation to a pre-cue baseline (Ojala and Bach, 2020; Lonsdorf et al., 2017). In the specific context of this report, we did not want to restrict analyses to discrete phasic responses and thus used the mean SCL signal in each bin as a measure of physiological response magnitude. Further, we wished to avoid relying on a pre-cue baseline which, by definition, includes a pre-encounter anticipation period, and thus used the tonic element of SCL in each bin as an individual-level baseline to diminish inter-individual differences in signal levels. Tonic levels change slowly: their magnitude did not change by bin, *p* = 0.50, or group, *p* = 0.51. Together, this analytic approach enabled us to capture any changes in physiological response at the bin level while avoiding the use of pre-cue baselines. Each bin response was then square-root transformed (Lonsdorf et al., 2017); following transformation, these values did not deviate from the normal distribution (*p*s > 0.16, Kolmogorov-Smirnov test). The four bins then comprised the factor of outcome imminence.

To facilitate interpretation, we first considered anxiety as a categorical variable (healthy comparisons [HC], anxiety patients); we then extended analyses to test dimensional effects of anxiety severity across the sample. First, we examined whether anxiety was associated with distinct temporal patterns of threat-anticipatory response with a repeated-measures ANOVA testing the effect of Cue × Imminence × Group on physiological response, with Cue (safety, threat) and Imminence (bins 1–4) as within-subject factors, and Group (HC, anxiety) as a between-subject factor. In line with our hypotheses, we then examined anxiety effects on two specific imminence-derived anticipatory physiological response measures: 1) initial response to threat (bin 1), and 2) magnitude of threat-specific *increase* in physiological response across the anticipation window (quantified as response in bin 4 minus response in bin 1, in threat relative to safety trials^1^). These indices were then used in the rsFC analyses (see below).

Significant interactions were decomposed by lower-order tests. Correlations reflect Pearson correlation coefficients. All tests were two-tailed; significance was set at *p* < 0.05. Cohen's *d* (*t*-tests) and partial eta squared (ANOVA) were used to calculate effect sizes.

### 2.1 Intrinsic connectivity correlates of anticipatory physiological response

Work in animals consistently links threat-anticipatory defensive responses to specific brain circuitry (Adolphs, 2013; Mobbs et al., 2020; Mobbs, 2018; Lefler et al., 2020; Perusini and Fanselow, 2015; Tovote et al., 2015; LeDoux, 2012; Fanselow and Dong, 2010). Key elements in this circuitry include subcortical structures such as basolateral amygdala (BLA), central nucleus of the amygdala (CeA), ventral (anterior in humans) hippocampus, bed nucleus of stria terminalis (BNST), nucleus accumbens (NAcc), midline thalamic nuclei (including the paraventricular nucleus (Kirouac, 2021)), lateral hypothalamus, and periaqueductal gray (PAG), as well as cortical regions, primarily infralimbic and prelimbic cortices (comparable to vmPFC and dorsal anterior cingulate/dorsomedial mid-cingulate prefrontal cortex, respectively), and anterior insula. Research in humans is beginning to extend such findings, indicating the involvement of these regions, as well as dorsolateral prefrontal cortex (dlPFC), in threat anticipation states (Mobbs et al., 2007, 2020; Roelofs, 2017; Robinson et al., 2019; Grupe and Nitschke, 2013; McNaughton and Corr, 2004; McMenamin et al., 2014; Andrzejewski et al., 2019; Chavanne and Robinson, 2021; Morriss et al., 2021). However, direct linking of function in this circuitry to physiological or behavioral indices of defensive responding in humans, as is done in animals, remains limited (Wendt et al., 2017; Terburg et al., 2018; McMenamin et al., 2014; Mobbs et al., 2007; Hermans et al., 2013; Suarez-Jimenez et al., 2018). Importantly, research linking perturbed function in this circuitry to aberrant defensive responding in anxiety is needed to establish its pathophysiological role. Here, we begin to bridge this gap by identifying intrinsic functional connectivity patterns that covary with individual differences in magnitude of threat-anticipatory physiological responses, and the moderation of such associations by anxiety.

**Imaging acquisition and preprocessing.** Resting state imaging data (10-min, eyes-open, multi-echo sequence; see supplement), including field map distortion correction scans, were acquired on a 3T MR750 General Electric scanner (Waukesha, Wisconsin, USA) at the NIMH in a different visit than the anticipation task (*med* = 27 days between visits; scan was performed prior to the task in 20 participants and after the task in 17 participants). Imaging data from 13 youths of the full sample were not available due to MRI contraindications or unavailability/refusal to scan (5 patients, 8 healthy controls). Functional imaging data were preprocessed and normalized to MNI space with FMRIPrep (Esteban et al., 2019); see supplement. Preprocessed data were then imported into CONN software (Whitfield-Gabrieli and Nieto-Castanon, 2012), where they were resampled into 2 mm resolution and underwent denoising which included removal (by means of regression) of pre-steady-state outliers, cosine, and all ICA-AROMA time-series regressors (Pruim et al., 2015), which is particularly suited for network identifiability (Ciric et al., 2017). Further, standard bandpass filter [0.008–0.09Hz] and linear detrending were applied. Quality control–functional connectivity (QC–FC) correlations for all motion variables indicated 90.7–98.6% overlap with null-hypothesis distribution, indicating effective motion denoising (Ciric et al., 2017). As suggested elsewhere (Kark et al., 2021; Alakorkko et al., 2017; Triana et al., 2020; Limbachia et al., 2021), unsmoothed data were used in analyses to avoid signal “spillage” between nearby regions of interest (ROIs) which could artificially affect connectivity measures. One participant was excluded from analyses due to excessive motion, leaving a total of 36 participants for rsFC analyses; see supplement for more details.

**Imaging data analysis.** We aimed to identify patterns of intrinsic functional connectivity within the defensive response circuitry (Limbachia et al., 2021) that relate to the magnitude of expressed threat-anticipatory physiological response, indexing preparation for execution of defensive responses (Fanselow et al., 1988; Hamm, 2019). We focused on two potential effects: the magnitude of *initial* response to threat cues (first bin) and the magnitude of *increase* in anticipatory response across the anticipation window (increase in response from first to last bin).

Given the conserved nature of this circuitry and to facilitate translational, cross-species research, the selection of structures to be included as subcortical ROIs in the defensive response network was based on the consistent findings from the extensive literature on defensive responding in rodents and primates (Adolphs, 2013; Fanselow et al., 1988; LeDoux, 2000; Mobbs et al., 2020; McNaughton and Corr, 2004; Perusini and Fanselow, 2015; Chavanne and Robinson, 2021). This selection is not biased by features of specific experimental human-subject paradigms. Accordingly, ROIs included (see Fig. 1B) bilateral BLA, CeA, anterior hippocampus, BNST, NAcc, midline thalamus, lateral hypothalamus, and PAG (single ROI). In the cortex, we included ROIs in bilateral vmPFC, dorsal anterior cingulate (dACC), dorsomedial PFC (dmPFC)/mid-cingulate, dlPFC, and anterior insula. All 25 ROIs in this network were defined using in-house scripts that merge several publicly-available, expert cortical and subcortical segmentations into common (MNI) standard space (available at: https://github.com/rany-abend/atlas). Thus, we used the segments as defined in these segmentations, rather than spheres which might artificially include heterogeneous regions; see supplement for more information on ROI selection and definition.

Once this network of 25 nodes was defined, an adjacency matrix of zero-order connectivity correlations between all node pairs (300 edges) was computed at the individual-subject level. Threat-anticipatory physiological response indices (from the task), anxiety severity (assessed continuously using SCARED scores, to increase power), and age (in years) were mean-centered and used as between-subject, second-level covariates. Network-Based Statistics (NBS) second-level analyses were then carried out, as implemented in CONN (Whitfield-Gabrieli and Nieto-Castanon, 2012; Zalesky et al., 2010), to pursue our second hypothesis. To identify specific subnetworks in which connectivity was associated with the magnitude of threat-anticipatory response, analyses tested the correlation between *network mass* (weighted sum of edges) and physiological response magnitude (Zalesky et al., 2010). We used an edge-wise initial uncorrected correlation threshold of *p* < 0.005 in conjunction with a threshold of *p*_*FDR*_<0.05 (Abend et al., 2019) for network mass; the test statistic was tested relative to 5000 permutations under the null hypothesis (Zalesky et al., 2010). Finally, to test our third hypothesis on anxiety moderation, we examined whether anxiety severity significantly moderated connectivity-response associations within this network, using a threshold of *p*_*FDR*_<0.05.

To complement edge-based analyses, we calculated *degree centrality* for each network node (number of edges connected to it). We then examined correlations between degree and physiological response magnitude to identify nodes that are particularly central within the network as it relates to response magnitude. Degree was calculated on adjacency matrices thresholded at *r* = 0.25 to avoid sparse connections. In all analyses, age was entered as a nuisance covariate.

## 3 Results

### 3.1 Threat-anticipatory physiological response and anxiety severity

**Fig. 2:**
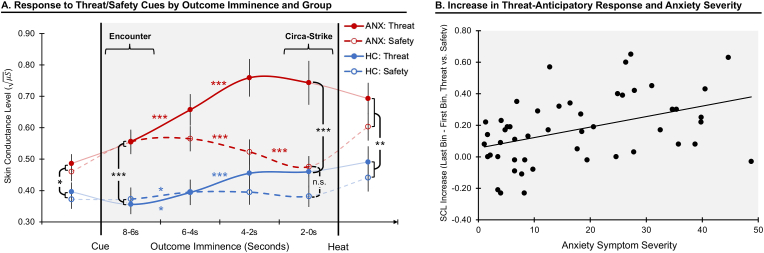
**Changes in anticipatory physiological response by threat imminence**. A) Area in gray represents anticipation period beginning with cue onset and up to imminent thermal stimulation. Lines reflect average skin conductance level in 2s bins in response to threat (highly painful heat, continuous lines) and safety (non-painful heat, dashed line), for the anxiety (red, ANX) and healthy control (blue, HC) groups. For completeness, we display responses prior to cue onset and during thermal stimulation (connected to the anticipation data in lighter lines). B) Scatterplot depicts association between anxiety symptom severity (assessed as a continuous measure) and magnitude of change in anticipatory physiological response to threat vs. safety (last bin minus first bin). *Note*: *, *p* < 0.05, **, *p* < 0.01, ***, *p* < 0.001. Colored asterisks reflect within-group effects; black asterisks reflect between-group effects. Change in response over time is represented as curved lines for descriptive purposes, to reflect its assumed continuous nature. (For interpretation of the references to colour in this figure legend, the reader is referred to the Web version of this article.)

Fig. 2A depicts mean anticipatory physiological response by cue, bin, and group (see Fig. S2 for individual-subject data). Analysis of physiological response during the 8s anticipation period indicated a significant Cue × Imminence interaction effect on SCL, *F*(3,144) = 35.15, *p* < 0.001, $\eta_{p}^{2}$ = 0.42, whereby the magnitude of SCL increased with imminence of the threat outcome, *F*(3,147) = 31.48, *p* < 0.001, $\eta_{p}^{2}$ = 0.39, and decreased with imminence of the safe outcome, *F*(3,147) = 6.33, *p* < 0.001, $\eta_{p}^{2}$ = 0.11. Across the sample, differential threat vs. safety responding significantly increased with outcome imminence, starting with absence of threat discrimination during initial response to the cues (bin 1), *t*(49) = 0.60, *p* = 0.55, *d* = 0.06, and reaching maximal differentiation during *circa-strike* (bin 4), *t*(49) = 5.70, *p* = 6.7 × 10^−7^, *d* = 0.88 (*t*-tests for dependent samples). Thus, the magnitude of anticipatory physiological response positively scaled, and showed greater threat specificity, with threat imminence.

This two-way interaction was qualified by a significant Cue × Imminence × Group interaction, *F*(3,144) = 7.55, *p* < 0.001, $\eta_{p}^{2}$ = 0.14, (Fig. 2A), indicating distinct temporal dynamics of anticipatory physiological responding as a function of anxiety. This interaction effect was decomposed in ways that considered our hypotheses. First, we examined anxiety effects on *initial* responses to threat cues (*encounter* phase, first bin following cue onset). We noted a significant effect of Group, *F*(1,48) = 17.05, *p* < 0.001, $\eta_{p}^{2}$ = 0.26, reflecting stronger initial responses across cues in the anxiety group. However, no Cue × Group effect was observed, *F*(1,48) = 0.45, *p* = 0.50, $\eta_{p}^{2}$ <0.01, indicating enhanced initial response to potential threat in anxiety prior to actual threat differentiation.

Second, we explicated anxiety differences in the unfolding of anticipatory response with threat imminence. In the anxiety group, a significant Cue × Imminence interaction was noted, *F*(3,72) = 32.37, *p* < 0.001, $\eta_{p}^{2}$ = 0.57, with follow-up analysis indicating that, relative to cue onset, response magnitude increased with threat imminence, *F*(3,72) = 21.55, *p* < 0.001, $\eta_{p}^{2}$ = 0.47, and decreased with imminence of the safe outcome, *F*(3,72) = 13.39, *p* < 0.001, $\eta_{p}^{2}$ = 0.36. During *circa-strike*, when the outcome was most imminent (bin 4), physiological responding in the anxiety group was significantly higher when threat relative to safety outcome was expected, *t*(24) = 6.68, *p* = 6.6 × 10^−7^, *d* = 1.33. In the HC group, we noted weaker response discrimination over time, *F*(3,72) = 6.22, *p* = 0.001, $\eta_{p}^{2}$ = 0.20, with response increasing in threat trials, *F*(3,72) = 11.63, *p* < 0.001, $\eta_{p}^{2}$ = 0.33, but not changing in safety trials, *F*(3,72) = 0.82, *p* = 0.49, $\eta_{p}^{2}$ = 0.03, and a modest imminent threat vs. safety difference during *circa-strike*, *t*(24) = 2.09, *p* = 0.048, *d* = 0.42. Comparing the groups during *circa-strike*, the anxiety group demonstrated a stronger response than the healthy group when anticipating immediate threat, *t*(48) = 3.79, *p* < 0.001, *d* = 1.08, but not safety, *t*(48) = 1.96, *p* = 0.056, *d* = 0.51.

Finally, as per our hypothesis, we quantified the magnitude of *increase* in threat-anticipatory response (last bin minus first bin in threat trials, relative to last bin minus first bin in safety trials). The anxiety group demonstrated a greater *threat-specific* increase in physiological response with imminence relative to the HC group, *F*(1,48) = 7.77, *p* = 0.008, $\eta_{p}^{2}$ = 0.14. The internal reliability of this index, assessed using Cronbach's alpha, was 0.77, indicating acceptable to good reliability. Together, anxiety was associated with a stronger initial response to cue onset, suggesting enhanced initial response to *potential* threat prior to threat discrimination. Anxiety was then associated with enhanced surge in anticipatory physiological response as threat became increasingly imminent.

To complement these group analyses, we examined whether the magnitude of initial response (bin 1) and evolving response (bin 4 minus bin 1) to anticipated threat was associated with individual differences in anxiety severity assessed continuously, in support of a dimensional mechanistic perturbation (Insel et al., 2010). Anxiety symptom severity (as measured using total SCARED scores) was positively correlated with initial response to threat cues, *r*(48) = 0.551, *p* < 0.001, and safety cues, *r*(48) = 0.437, *p* = 0.001, but not to threat vs. safety difference (difference in bin 1 response magnitudes), *r*(48) = 0.119, *p* = 0.41. In terms of increasing anticipatory response, anxiety symptom severity was positively correlated with increase in response during anticipation of threat, *r*(48) = 0.480, *p* < 0.001, but not safety, *r*(48) = 0.261, *p* = 0.07, with the difference between these correlation coefficients being significant, *Z* = 2.14, *p* = 0.016. Finally, we quantified threat-specific increase in anticipatory response (bin 4 minus bin 1 in threat trials, relative to bin 4 minus bin 1 in safety trials). The correlation between anxiety severity and threat-specific increase in response was significant, *r*(48) = 0.373, *p* = 0.008; see Fig. 2B. These results therefore replicate and extend the categorical analyses, indicating that anxiety along the severity continuum is associated with a threat-specific increase in anticipatory response with threat imminence. The threat-specific increase in response magnitude was then used in rsFC analyses (see below).

Of note, the anxiety-response correlation did not change when controlling for age, *r*(47) = 0.374, *p* = 0.008, or sex, *r*(47) = 0.387, *p* = 0.006. In the primary ANOVA analysis, we noted a Cue × Sex interaction on physiological response (irrespective of outcome imminence) which is reported in full in the supplement. However, the Cue × Imminence × Group effect of interest remained significant when controlling for age, *F*(3,141) = 7.36, *p* < 0.001, $\eta_{p}^{2}$ = 0.14, and sex, *F*(3,141) = 7.93, *p* < 0.001, $\eta_{p}^{2}$ = 0.14.

For completeness, we also report in the supplement on the Cue × Group effect during the period immediately before the cue-based anticipation window, and during thermal stimulation. Briefly, we observed a modestly greater pre-cue physiological response by the anxiety group (main effect). The primary interaction effect reported above (Cue × Imminence × Group) remained significant when controlling for pre-cue response. Response to thermal stimulation indicated greater response to the highly-painful heat relative to the non-painful heat (main effect), and a greater response by the anxiety group relative to the HC group (main effect), although this group effect was completely abolished once anticipatory response magnitude (bin 4) was covaried. See supplement for full details.

### 3.2 Intrinsic functional connectivity and threat-anticipatory physiological response

Imaging analyses focused on identifying network-level patterns of intrinsic functional connectivity that correlate with the magnitude of threat-anticipatory responding. Of note, threat-specific anxiety effects emerged only when quantifying the magnitude of *increase* in physiological response with threat imminence across the anticipation window (see above). As a result, we directed our analyses to identify rsFC correlates of this effect. To this end, we examined correlates of the threat-specific *increase* in anticipatory response, i.e., bin 4 minus bin 1 in threat trials relative to bin 4 minus bin 1 in safety trials, as defined above. rsFC associations with the magnitude of initial, non-threat-specific response are reported in Fig. S5.

**Fig. 3:**
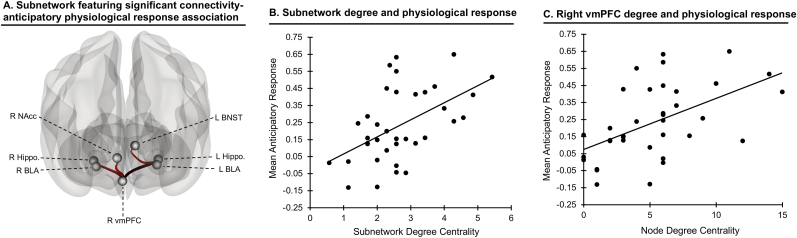
**Subnetwork associated with threat-anticipatory physiological response**. A) Functional connectivity within this subnetwork positively correlated with magnitude of increase in threat-anticipatory physiological response (*p*_*FDR*_<0.05). All depicted edges are positive. B) Significant association between the subnetwork's degree centrality (average connectedness of nodes within it) and mean anticipatory physiological response magnitude; greater network connectedness was associated with greater response. C) Significant association between right vmPFC degree centrality and mean anticipatory physiological response magnitude; greater vmPFC connectedness to other nodes was associated with greater response. *Note*: R = right, L = left, NAcc = nucleus accumbens, BNST = bed nucleus of the stria terminalis, BLA = basolateral amygdala, hippo = hippocampus, vmPFC = ventromedial prefrontal cortex.

Our primary analysis considered multivariate correlations among all edges in the defensive response network and physiological response magnitude, to identify subnetworks that most strongly relate to responding. This analysis revealed a specific subnetwork in which intrinsic connectivity significantly correlated with individual differences in response magnitude at the prespecified threshold, network *mass* = 140.20, *p*_*FDR*_ = 0.035; see Fig. 3A (also see Fig. S4 for all edges at a lower significance threshold). The extent of connectivity within this subnetwork as assessed by its overall degree centrality (average connectedness of nodes within it) was likewise associated with individual differences in physiological responding, *t*(33) = 2.35, *p*_*FDR*_ = 0.025 (Fig. 3B). This subnetwork comprised positive cortical-subcortical connectivity-response edges, including right vmPFC connections with bilateral BLA (*p*s < 0.004), bilateral hippocampus (*p*s < 0.001), and right NAcc (*p* = 0.003), as well as positive left hippocampus-BNST connectivity (*p* = 0.004). Among network nodes, right vmPFC demonstrated the strongest association between node degree and physiological response, *t*(33) = 4.32, *p*_*FDR*_<0.001 (Fig. 3C).

Finally, we examined moderation of connectivity-physiological response associations by anxiety severity in this subnetwork, using NBS and the same statistical threshold as above. A three-edge subnetwork, including right vmPFC links with bilateral hippocampus and left BLA, demonstrated a significant anxiety moderation effect of the association between connectivity and physiological response magnitude, *p*_*FDR*_ = 0.005, such that greater anxiety was associated with stronger connectivity-response correlation (Fig. S6).

Additional auxiliary analyses verified that the connectivity effects identified above did not reflect general covariation with non-specific physiological responsivity. Network analyses using the specified thresholds did not detect any networks in which connectivity was significantly associated with magnitude of physiological response during pre-cue (*pre-encounter*) or thermal stimulation, all *p*_*FDRs*_>0.05. To further examine specificity of findings, no significant associations emerged when threat imminence was not considered, i.e., when physiological response was averaged across bins in threat trials, or when the difference in averaged response in threat vs. safety trials was used in analysis, *p*_*FDRs*_>0.05. Thus, only when considering threat imminence did associations emerge between physiological response and function in the defensive response network.

## 4 Discussion

This report incorporates insight from translational research on fear to characterize aberrant dynamics of threat-anticipatory physiological responding in anxiety. Analyses revealed three primary imminence-dependent findings. First, anxiety severity was positively associated with magnitude of physiological response to onset of both threat and safety cues. Second, anxiety moderated the temporal unfolding of anticipatory physiological response; with increasing imminence of physical threat, greater anxiety severity was associated with stronger anticipatory response. Finally, the magnitude of increase in threat-anticipatory response corresponded to intrinsic functional connectivity within a conserved cortical-subcortical circuit, with vmPFC constituting a central node; importantly, more severe anxiety was associated with a stronger association between magnitude of increase in anticipatory response and magnitude of connectivity that the vmPFC showed with BLA and hippocampus. Together, these findings link anxiety severity to unique temporal patterns of threat-anticipatory physiological response and variations in cortical-subcortical intrinsic functional connectivity.

Anxiety symptoms are clinically characterized by excessive defensive responding in anticipation of threat (Corchs and Schiller, 2019; American Psychiatric Asso, 2013). Translational research demonstrates anxiety-related enhanced physiological responses to potential threat during threat anticipation (Grupe and Nitschke, 2013; Lissek et al., 2005; Grillon, 2002; Grillon et al., 2019; Dvir et al., 2019; Lonsdorf and Merz, 2017). Our recent work further shows that excessive physiological responding in anxiety is specific to anticipation, but not ultimate experience, of aversive stimuli (Abend et al., 2020b, 2021). Such work establishes links between aberrant threat anticipation processes and anxiety symptoms but does not consider threat imminence. Indeed, animal research suggests that an important determinant of defensive responding is proximity to threat. Research in healthy humans begins to extend this work, confirming that dynamic changes in physiological responding and defensive behaviors occur as a function of threat imminence. Given that threat-anticipatory defensive responding is central in the presentation of anxiety (American Psychiatric Asso, 2013; Abend et al., 2020a), identifying these response dynamics could more precisely inform on psychopathological mechanisms. This is the first study to map dynamic patterns of imminence-induced changes in defensive responding to anxiety symptoms.

The primary effect that we observed was increasing physiological response as threat became more imminent, in line with prior research (Hamm, 2019; Mobbs, 2018; Low et al., 2008; Wendt et al., 2017; Mobbs et al., 2007). In the context of defensive responding, this anticipation window corresponds to *post-encounter* to *circa-strike* phases, and it is associated with acute responding: physiological response discharge and increased execution of active defensive behaviors. Indeed, we observed an increase in physiological response as highly-painful, but not non-painful, thermal stimulation became more imminent. Importantly, we extend such basic-science and translational research by demonstrating that anxiety moderates this effect. Specifically, relative to safety, greater evolving response to threat was observed in anxiety, culminating in a maximal anxiety effect during threat *circa-strike*. These findings highlight links between anxiety severity and perturbations in the mechanisms generating acute responding with increasing threat imminence.

Extensive research in animals identifies the brain circuitry involved in threat-imminent defensive responding by linking functional modulation in specific structures and changes in defensive behaviors (Adolphs, 2013; Mobbs et al., 2020; Mobbs, 2018; Choi and Kim, 2010; Lefler et al., 2020; Amorapanth et al., 2000). A distributed circuit comprising vmPFC, BLA, and hippocampus has been shown to promote the expression of excessive fear responses and anxiety-like behaviors in animals (Hagihara et al., 2021; Herry et al., 2008; Adhikari et al., 2010; Felix-Ortiz et al., 2013; Tye et al., 2011; Padilla-Coreano et al., 2016; Likhtik et al., 2014). The involvement of these structures in the expression of defensive responses, including physiological responses, has recently been extended to humans (Qi et al., 2018; Wendt et al., 2017; Terburg et al., 2018; Myers-Schulz and Koenigs, 2012; Schiller and Delgado, 2010; Mobbs et al., 2007; Chavanne and Robinson, 2021; Suarez-Jimenez et al., 2018; Fung et al., 2019; Wager et al., 2009; Zhang et al., 2014), and suggests that such responding depends on orchestrated multi-structure function. Here, we extend prior work in three important ways. By using network analyses, we characterize the multivariate nature of this circuit's function as it corresponds to the expression of physiological response to threat. Second, we link functional variation in this circuit to anxiety severity, supporting translational conceptualizations implicating aberrant function of the vmPFC-BLA-hippocampus circuit in dysregulated fear responses in anxiety (Mobbs, 2018; Felix-Ortiz et al., 2013; Padilla-Coreano et al., 2016; Milad and Quirk, 2012; Moscarello and Maren, 2018). Third, effects emerged on resting-state connectivity patterns collected separately from the task, indicating a potential bias in this network's intrinsic function that is clinically meaningful (Deco and Jirsa, 2012).

Within this circuit, vmPFC centrality related most strongly to physiological response magnitude. Considerable research in humans and animals highlights the role of vmPFC in threat processing and fear/anxiety states (Schiller and Delgado, 2010; Amorapanth et al., 2000; Milad and Quirk, 2012; Likhtik and Paz, 2015; Ramirez et al., 2015). Likewise, vmPFC-ventral hippocampus connectivity has been implicated in the expression of fear responses (Adhikari et al., 2010; Padilla-Coreano et al., 2016; Lesting et al., 2011). More broadly, a recent conceptualization implicates posterior vmPFC in threat (but not safety) assessment (Tashjian et al., 2021). Indeed, we show that connectivity with this region was correlated with magnitude of response to threat. Some suggest that vmPFC exerts top-down response regulation in this circuit (Myers-Schulz and Koenigs, 2012; Milad and Quirk, 2012; Motzkin et al., 2015), while others propose that vmPFC maintains fear representations potentially driven by bottom-up innervation (Myers-Schulz and Koenigs, 2012; Padilla-Coreano et al., 2016; Aggleton et al., 2015). Our observation of a positive association between connectivity-response correlation and anxiety severity, coupled by predominantly positive connectivity coefficients, is perhaps more in line with the latter notion (Myers-Schulz and Koenigs, 2012), although additional research is needed for more conclusive inferences.

BNST-hippocampus connectivity was part of the subnetwork in which function correlated with physiological responding. BNST has been implicated as a key structure in defensive responding (Fox and Shackman, 2019; Robinson et al., 2019), with studies demonstrating its function in response to both imminent and sustained threat (Davis et al., 2010; Straube et al., 2007; Mobbs et al., 2010). Specifically, BNST-hippocampus connectivity has been suggested to modulate fear responding by integrating contextual information (Goode and Maren, 2017). Here, increasing threat imminence may potentially reflect a contextual shift as threat is increasingly temporally proximal, invoking this functional link. Additionally, we identified vmPFC-NAcc connectivity in the subnetwork. NAcc is ascribed a central role in mediating the behavioral aspects of defensive responses in face of imminent threat, and active avoidance in particular (Moscarello and Maren, 2018; Ramirez et al., 2015; Levita et al., 2012). Thus, enhanced NAcc connectivity may suggest an increased propensity to execute acute defensive behaviors with increasing threat imminence. Interestingly, while research identifies CeA and PAG as outputs of this network in generation of defensive responses (Mobbs et al., 2007, 2020; Tye et al., 2011), these did not significantly emerge in our data. This might suggest that individual differences in defensive responding tendencies manifest primarily as variation in function of upstream network components in which inputs from multiple structures converge to influence the selection and maintenance of defensive responses, rather than in network effectors mediating their immediate execution (Adolphs, 2013).

Contrary to our hypothesis, anxiety severity was not associated with greater threat-specific initial response to cue onset. In the context of potential encounter with threat, cue onset may correspond to the *encounter* phase (or start of the *post-encounter* phase)(Mobbs, 2018). Anxiety was rather associated with elevated physiological response that appears to precede the discrimination of threat vs. danger. Considering that physiological responding facilitates defensive behaviors, this enhanced response could reflect a drive to carry out freezing or passive avoidance behavior with cue onset, enabling initial risk assessment (Ojala and Bach, 2020). Only following initial assessment of potential threat value and as outcomes became increasingly imminent, did physiological response show threat discrimination. Considering threat imminence chronometry, cue onset occurs against the backdrop of a *pre-encounter* phase, in which subjects await the next trial. It is possible that in the context of *pre-encounter*, the onset of any stimulus that may predict danger initiates in individuals with anxiety a stronger risk assessment response. Indeed, these findings are in line with our recent work showing that anxiety is associated with enhanced physiological response to any cue that could potentially predict danger (Abend et al., 2020b).

Extensive research attempts to identify pathophysiological markers for psychiatric disorders which could promote improved prediction, diagnosis, and treatment (FitzGerald, 2016; Garcia-Gutierrez et al., 2020; Xia and Kheirbek, 2020). The increase in physiological response to threat identified here could provide initial indications for a potential anxiety biomarker, and encourage continued research for a number of reasons. First, threat-specific increase in anticipatory physiological response differentiated the healthy and anxiety groups. Second, increased physiological response provides face validity for this potential biomarker as acute physiological response to imminent is central in the presentation of anxiety symptoms (American Psychiatric Asso, 2013; Abend et al., 2020a); further, this increase in response showed adequate internal consistency. Third, the association between response and anxiety severity also manifested dimensionally, in line with recent conceptualizations of psychopathology (Insel et al., 2010), potentially offering greater sensitivity for identifying at-risk, sub-threshold individuals (Xia and Kheirbek, 2020). Fourth, we linked this index to specific patterns of functional connectivity that correspond to findings from animal research; this could promote continued cross-species research on circuit-based biomarkers (Xia and Kheirbek, 2020). Finally, we observed these effects in youth with only anxiety disorders; this suggests specificity as well as potential utility for developmental samples. While these findings encourage continued research, additional work is required, including replication and continued assessment of reliability, validity, and specificity.

More broadly, our findings could be considered in the context of conceptualizations of affective chronometry, linking temporal dynamics of emotional responding to well-being and psychopathology (Davidson, 1998, 2015; Lapate and Heller, 2020). For example, prolonged endocrine and physiological responding following stressful events and processing of negative stimuli has been linked to depressive symptoms and other traits associated with reduced well-being (Burke et al., 2005; Javaras et al., 2012). In animal and human models of anxiety, sustained freezing behavior when encountering threat is associated with risk for pathological anxiety (Fox and Kalin, 2014). Here, we add to this literature by demonstrating how the incorporation of the dimension of time reveals unique links among threat-anticipatory physiological responding, intrinsic brain function, and anxiety. Continued research on the chronometry of threat anticipation could further provide clues on how biological responses and subjective experiences of fear are related, and potentially identify intervention targets (Davidson, 2015).

Several important limitations should be considered. First, sample size was modest; while effects were generally robust and emerged using continuous measures, a replication is nonetheless advised. Second, we did not study youth with disorders other than anxiety; such samples are needed to establish the specificity of effects more conclusively. Third, imaging analyses relied on a 3T scanner; future work using higher fields could provide better image resolution and delineation of smaller subcortical structures relevant to the processes of interest. Fourth, future research should add additional physiological measures as well as assess continuous fear/anxiety reports throughout the task and measure defensive behaviors (Roelofs, 2017). Finally, given neural and hormonal maturation during adolescence and early adulthood, replication of these findings in adults with anxiety is warranted.

## 5 Conclusions

This report links translational research on fear, clinical research in anxiety patients, and physiological and neuroimaging research, to link aberrant patterns of threat imminence-dependent anticipatory responding and anxiety severity. By considering threat imminence, we quantify a physiological index of anticipatory defensive responding that robustly differentiates anxious and healthy individuals and has intrinsic functional connectivity correlates that correspond to findings from animal studies on defensive responding circuitry. These findings advance our understanding of normative and abnormal threat-anticipatory fear responses and establish a potential biomarker for anxiety.

## CRediT authorship contribution statement

**Rany Abend:** Conceptualization, Methodology, Validation, Formal analysis, Investigation, Data curation, Writing – original draft, Writing – review & editing, Visualization, Supervision, Project administration. **Sonia G. Ruiz:** Methodology, Validation, Formal analysis, Data curation, Writing – original draft, Writing – review & editing, Visualization. **Mira A. Bajaj:** Methodology, Validation, Formal analysis, Data curation, Writing – original draft, Writing – review & editing. **Anita Harrewijn:** Investigation, Data curation, Writing – original draft, Writing – review & editing. **Julia O. Linke:** Methodology, Investigation, Writing – original draft, Writing – review & editing. **Lauren Y. Atlas:** Methodology, Formal analysis, Investigation, Writing – original draft, Writing – review & editing. **Anderson M. Winkler:** Methodology, Formal analysis, Resources, Software, Writing – review & editing. **Daniel S. Pine:** Resources, Writing – original draft, Writing – review & editing, Supervision, Project administration, Funding acquisition.

## Declaration of competing interest

The authors declare that they have no known competing financial interests or personal relationships that could have appeared to influence the work reported in this paper.

## Footnotes

1 Of note, this quantification measure correlated very highly, *r*_*s*_ > 0.90, *p*_*s*_ < 3.9 × 10^−19^, with alternative related indices, such as difference between slopes of change and difference between raw responses in bin 4. Thus, these parallel measures that capture the increase in response are equivalent.
