# Supplementary material for: Threat imminence reveals links among unfolding of anticipatory physiological response, cortical-subcortical intrinsic functional connectivity, and anxiety
Source: Neurobiol Stress. 2022 Jan 4;16:100428. doi: 10.1016/j.ynstr.2022.100428 (PMC8749274; doi:10.1016/j.ynstr.2022.100428)
Supplement: Multimedia component 1 [file mmc1.docx]

**Supplement**

**Participants**

As we reported previously^1^, this study recruited 64 youths from the community, including 33 treatment-seeking anxiety patients and 31 healthy youth. Data were excluded for participants who did not show reliable pain rating during calibration (see below; 2 patients, 2 healthy); aborted the task (5 patients, 1 healthy); or technical problems in data collection (1 patient, 3 healthy). The distribution of SCARED scores for the sample is presented in Fig. S1.

**Exclusion criteria**. Exclusionary criteria included IQ<70^2^, diagnosis of autism spectrum disorder, posttraumatic stress disorder, schizophrenia, attention-deficit hyperactivity disorder, major depressive disorder, obsessive compulsive disorder, or neurological disorders, history of trauma, recent use of psychoactive substances, or significant medical illness. Patients were required to meet criteria for generalized, social, and/or separation anxiety disorder. Healthy participants did not meet criteria for any psychiatric diagnosis.


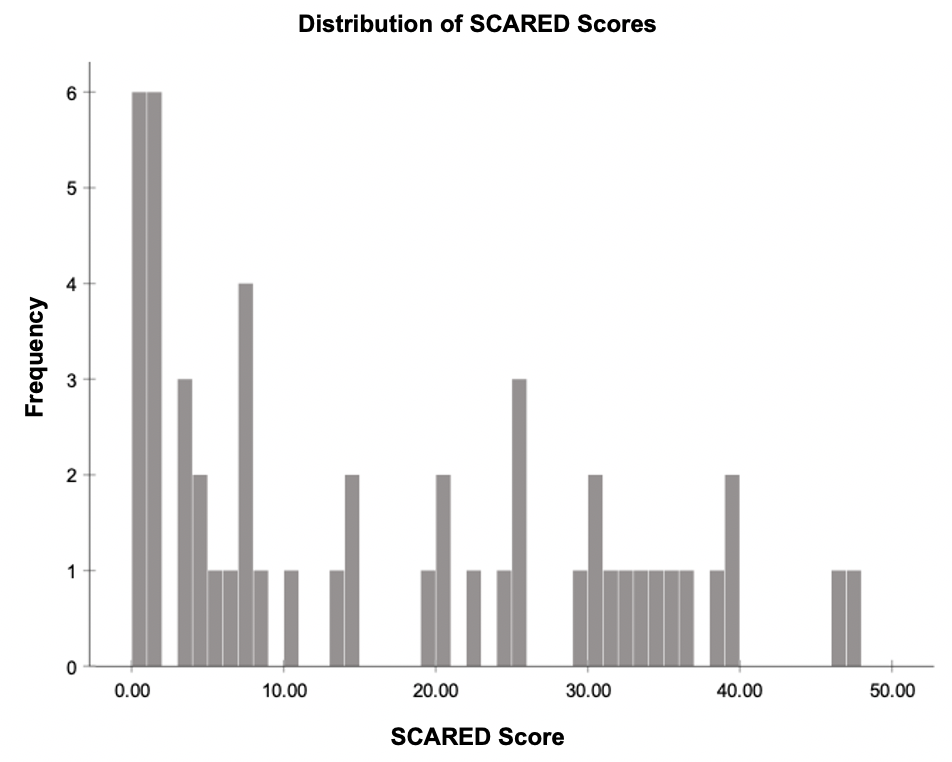


**Figure S1.** Distribution of the Screen for Child Anxiety Related Emotional Disorders (SCARED) scores for the sample used in analyses.

**Anticipation task**

**Thermal stimulation and calibration**. Stimulation followed prior procedures^3,4^, and details have also been published in our prior report using this sample^1^. Stimulation was delivered to left forearm using a 27mm diameter CHEPS thermode (Medoc Ltd, Ramat-Yishai, Israel). Each stimulation lasted 5 seconds (one per trial), including 0.5-second ramp-up, 4 seconds at target temperature, and 0.5-second ramp-down. Immediately prior to the task, participants completed an adaptive staircase pain calibration^3,4^. Possible temperatures to be delivered ranged from 34°C to 48°C, in increments of 0.5°C. Participants rated subjective pain (0-10) following each pain delivery using the FACES pain scale^5^. To start the calibration procedure, two initial temperatures of 34°C and 36°C were applied to the right arm to acclimate participants to the procedure. Next, stimulation was applied to four skin sites over 18 trials. The three sites with the highest rating accuracy (lowest average residuals from linear regression predicting pain by temperature) were used in the task. Ratings were used to estimate temperatures that would reliably elicit subjective-pain ratings of 2 (low-pain), 5 (moderately-painful), and 8 (highly-painful). Participants were required to show reliable temperature-ratings correlation (R^2^≥0.40)^3,4^ to start the task. Healthy and patient groups did not significantly differ in temperature (degrees Celsius) corresponding to low pain (level 2; M[SD]=38.20 [2.12] and M[SD]=37.2 [2.51], respectively), medium pain (level 5; M[SD]=42.32 [1.86] and M[SD]=40.90 [2.89], respectively), or high pain (level 8; M[SD]=46.28 [2.30] and M[SD]=45.58 [2.11], respectively). Groups also did not differ in reliability of temperature-pain relationship (R^2^; M[SD]=0.72 [0.13] and M[SD]=0.75 [0.14], respectively), all *p*s>0.05. Thus, comparable levels of pain across the sample were used in the task.

**Threat anticipation task**. The anticipation induction task consisted of visual shape stimuli (yellow triangle, blue square) paired with thermal stimulation. At the start of the task, participants were instructed that one shape predicts low-pain thermal stimulation (corresponding to level-2 pain rating) while the other predicts highly-painful thermal stimulation (level-8 pain). Shape-pain assignment was counterbalanced across participants. To verify that participants understood the conditions, we collected expected pain ratings at the start of each block. These indicated that participants expected more pain following the high-pain cue than the low-pain cue, *F*(1,448)=2091.96, *p*<0.001; groups did not differ in these ratings.

In each trial (Fig. 1, main text), participants were presented with either the low-pain or high-pain cue (2 seconds). Eight seconds after cue onset, thermal stimulation was delivered (low-, moderate-, or high-pain temperature; 5 seconds). After a variable delay (5-7 seconds), participants used a mouse to provide pain-ratings (0-10); see^1^. Variable inter-trial interval (4-6 seconds) separated trials. The task comprised 36 trials of interest which included combinations of low-pain cue followed by low-pain temperature (LL) and high-pain cue followed by high-pain temperature (HH). In addition, the original task also included trials where low-pain and high-pain cues were followed by the moderate-pain temperature (corresponding to level-5 pain; LM, HM), to examine anticipatory effects on pain experience, which are not reported here; see^1^. Trials were delivered in 8 blocks; at the start of each block, the experimenter moved the thermode to a different arm location.

**Imaging acquisition**

Resting state imaging data were acquired on a 3.0 Tesla General Electric scanner using a 32-channel head coil. Blood-oxygen-level-dependent (BOLD) signal was measured during the 10-minute scan with a multi-echo planar imaging sequence (TR: 2000 msec, TE1/2/3: 14.8/28.4/42.0 msec, respectively; flip angle: 77°; FoV: 240x240x260; matrix size: 64×64; 34 axial interleaved slices; slice thickness: 3.8mm; bandwidth: 7812.5 Hz/Pixel). During the scan, participants were instructed to keep their eyes open and focus on white fixation cross projected on the middle of a black screen. Additionally, a high-resolution T1-weighted, magnetization prepared, rapid-acquisition gradient echo (MPRAGE) sequence was acquired (TE: min full; TI: 425; FoV: 25.6; freq×phase: 256×256; flip angle: 7°; 1×1×1 mm voxels).

**Imaging preprocessing**

Imaging data were preprocessed using fMRIPrep. The following text was generated by the program is provided here verbatim to improve replicability.

Results included in this manuscript come from preprocessing performed using fMRIPrep 20.2.1^6,7^ (RRID:SCR_016216), which is based on Nipype 1.5.1^8,9^ (RRID:SCR_002502).

**Anatomical data preprocessing.** A total of 1 T1-weighted (T1w) images were found within the input BIDS dataset. The T1-weighted (T1w) image was corrected for intensity non-uniformity (INU) with N4BiasFieldCorrection^10^, distributed with ANTs 2.3.3^11^ (RRID:SCR_004757), and used as T1w-reference throughout the workflow. The T1w-reference was then skull-stripped with a Nipype implementation of the antsBrainExtraction.sh workflow (from ANTs), using OASIS30ANTs as target template. Brain tissue segmentation of cerebrospinal fluid (CSF), white-matter (WM) and gray-matter (GM) was performed on the brain-extracted T1w using fast FSL 5.0.9^12^ (RRID:SCR_002823). Brain surfaces were reconstructed using recon-all FreeSurfer 6.0.1^13^ (RRID:SCR_001847), and the brain mask estimated previously was refined with a custom variation of the method to reconcile ANTs-derived and FreeSurfer-derived segmentations of the cortical gray-matter of Mindboggle^14^ (RRID:SCR_002438). Volume-based spatial normalization to two standard spaces (MNI152NLin2009cAsym, MNI152NLin6Asym) was performed through nonlinear registration with antsRegistration (ANTs 2.3.3), using brain-extracted versions of both T1w reference and the T1w template. The following templates were selected for spatial normalization: ICBM 152 Nonlinear Asymmetrical template version 2009c [^15^ (RRID:SCR_008796; TemplateFlow ID: MNI152NLin2009cAsym)], FSL’s MNI ICBM 152 non-linear 6th Generation Asymmetric Average Brain Stereotaxic Registration Model [^16^ (RRID:SCR_002823; TemplateFlow ID: MNI152NLin6Asym)].

**Functional data preprocessing.** For each of the 1 BOLD run found per subject, the following preprocessing was performed. First, a reference volume and its skull-stripped version were generated from the shortest echo of the BOLD run using a custom methodology of fMRIPrep. A B0-nonuniformity map (or fieldmap) was estimated based on two (or more) echo-planar imaging (EPI) references with opposing phase-encoding directions, with 3dQwarp^17^ (AFNI 20160207). Based on the estimated susceptibility distortion, a corrected EPI (echo-planar imaging) reference was calculated for a more accurate co-registration with the anatomical reference. The BOLD reference was then co-registered to the T1w reference using bbregister (FreeSurfer) which implements boundary-based registration^18^. Co-registration was configured with six degrees of freedom. Head-motion parameters with respect to the BOLD reference (transformation matrices, and six corresponding rotation and translation parameters) are estimated before any spatiotemporal filtering using mcflirt^19^ (FSL 5.0.9). BOLD runs were slice-time corrected using 3dTshift from AFNI 20160207^17^. The BOLD time-series (including slice-timing correction when applied) were resampled onto their original, native space by applying a single, composite transform to correct for head-motion and susceptibility distortions. These resampled BOLD time-series will be referred to as preprocessed BOLD in original space, or just preprocessed BOLD. A T2* map was estimated from the preprocessed BOLD by fitting to a monoexponential signal decay model with nonlinear regression, using T2*/S0 estimates from a log-linear regression fit as initial values. For each voxel, the maximal number of echoes with reliable signal in that voxel were used to fit the model. The calculated T2* map was then used to optimally combine preprocessed BOLD across echoes following the method described in ^20^. The optimally combined time series was carried forward as the preprocessed BOLD. First, a reference volume and its skull-stripped version were generated using a custom methodology of fMRIPrep. The BOLD time-series were resampled onto the following surfaces (FreeSurfer reconstruction nomenclature): fsaverage. The BOLD time-series were resampled into standard space, generating a preprocessed BOLD run in MNI152NLin2009cAsym space. First, a reference volume and its skull-stripped version were generated using a custom methodology of fMRIPrep. Automatic removal of motion artifacts using independent component analysis^21^ (ICA-AROMA) was performed on the preprocessed BOLD on MNI space time-series after removal of non-steady state volumes and spatial smoothing with an isotropic, Gaussian kernel of 6mm FWHM (full-width half-maximum). Corresponding “non-aggresively” denoised runs were produced after such smoothing. Additionally, the “aggressive” noise-regressors were collected and placed in the corresponding confounds file. Several confounding time-series were calculated based on the preprocessed BOLD: framewise displacement (FD), DVARS and three region-wise global signals. FD was computed using two formulations following Power (absolute sum of relative motions)^22^ and Jenkinson (relative root mean square displacement between affines)^19^. FD and DVARS are calculated for each functional run, both using their implementations in Nipype (following the definitions by Power). The three global signals are extracted within the CSF, the WM, and the whole-brain masks. Additionally, a set of physiological regressors were extracted to allow for component-based noise correction (CompCor)^23^. Principal components are estimated after high-pass filtering the preprocessed BOLD time-series (using a discrete cosine filter with 128s cut-off) for the two CompCor variants: temporal (tCompCor) and anatomical (aCompCor). tCompCor components are then calculated from the top 2% variable voxels within the brain mask. For aCompCor, three probabilistic masks (CSF, WM and combined CSF+WM) are generated in anatomical space. The implementation differs from that of Behzadi et al. in that instead of eroding the masks by 2 pixels on BOLD space, the aCompCor masks are subtracted a mask of pixels that likely contain a volume fraction of GM. This mask is obtained by dilating a GM mask extracted from the FreeSurfer’s aseg segmentation, and it ensures components are not extracted from voxels containing a minimal fraction of GM. Finally, these masks are resampled into BOLD space and binarized by thresholding at 0.99 (as in the original implementation). Components are also calculated separately within the WM and CSF masks. For each CompCor decomposition, the k components with the largest singular values are retained, such that the retained components’ time series are sufficient to explain 50 percent of variance across the nuisance mask (CSF, WM, combined, or temporal). The remaining components are dropped from consideration. The head-motion estimates calculated in the correction step were also placed within the corresponding confounds file. The confound time series derived from head motion estimates and global signals were expanded with the inclusion of temporal derivatives and quadratic terms for each^24^. Frames that exceeded a threshold of 0.5 mm FD or 1.5 standardised DVARS were annotated as motion outliers. All resamplings can be performed with a single interpolation step by composing all the pertinent transformations (i.e. head-motion transform matrices, susceptibility distortion correction when available, and co-registrations to anatomical and output spaces). Gridded (volumetric) resamplings were performed using antsApplyTransforms (ANTs), configured with Lanczos interpolation to minimize the smoothing effects of other kernels^25^. Non-gridded (surface) resamplings were performed using mri_vol2surf (FreeSurfer).

Many internal operations of fMRIPrep use Nilearn 0.6.2 (RRID:SCR_001362)^26^, mostly within the functional processing workflow. For more details of the pipeline, see the section corresponding to workflows in fMRIPrep’s documentation.

**Copyright Waiver.** The above boilerplate text was automatically generated by fMRIPrep with the express intention that users should copy and paste this text into their manuscripts unchanged. It is released under the CC0 license.

**Imaging analysis: Removing outliers**

Outlier detection was performed in CONN. One subject deviated by more than 1.5 inter-quartile range values from the first or third quartiles in number of usable volumes (QC_ValidScans), maximal motion (QC_MaxMotion), mean motion (QC_MeanMotion), and maximal global signal change (QC_MaxGSchange), and was thus removed from resting-state analyses, leaving a total sample of 36 participants. Anxiety severity was not significantly associated with any of these parameters, *t*(35)s≤1.20, *p*s≥0.240.

**Imaging analysis: ROI selection and merging**

**ROI selection**. Regions of interest (ROIs) for the imaging analyses were selected based on the extensive literature implicating their involvement in defensive responding. While individual studies suggest the involvement of additional regions in such processes, here we took the subgroup of subcortical and cortical structures that is consistently implicated in defensive responding. These form an interconnected network whose specific characterization is the subject of intensive research in animals, particularly as it relates to the assessment of risk/safety and selection and execution of defensive behaviors^27-30^. It is not yet clear how function within this network translates to humans. Here, we begin to characterize this network’s function as it relates to the variable expression of defensive responses. The emphasis on translational research as a source for defining these ROIs is to facilitate continued cross-species research.

To define these ROIs, we used in-house scripts that merge defined regions from different expert segmentations into a single standard space (MNI volumetric space) while removing overlapping voxels. Complete details, code, and links to original segmentations are available at: <https://github.com/rany-abend/atlas>.

Basolateral amygdala (BLA) has been consistently implicated in the selection and execution of fear and defensive responses, potentially by integration of multiple inputs about the threat^29,31^; it was defined by the union of the basal nucleus, lateral nucleus, and accessory basal nucleus extracted from the amygdala subnuclei segmentation, available with the FreeSurfer pipeline^32^, that was run on the high-resolution mni_icbm152_nlin_asym_09b template (http://www.bic.mni.mcgill.ca/~vfonov/icbm/2009/mni_icbm152_nlin_asym_09b_nifti.zip). The central nucleus of the amygdala is consistently linked to the expression of defensive behaviors, for example via innervation of periaqueductal gray^29,33,34^, and was similarly derived, but included the central and medial nuclei. The hippocampus was derived from the hippocampal subfield segmentation available with the FreeSurfer pipeline^35^, and included all structures within the Head grouping. Ventral (comparable to human anterior) hippocampus plays a key role in defensive responding by regulating stress responses and conveying information on environmental threat/safety^27,36^. The bed nucleus of the stria terminalis (BNST) is strongly implicated in threat-anticipation states, partly via connectivity with hippocampus and midline thalamus, by regulating functions such as stress response^37,38^; here, it was derived from an expert segmentation of the hypothalamus^39^. Midline thalamic nuclei also play a key role in defensive responding, at least partly via connections to BLA and medial prefrontal cortex^28,40^. Here, they were derived from the thalamic segmentation available with the FreeSurfer pipeline ^41^, and included the medial and lateral mediodorsal nuclei and nucleus reuniens; this region also includes the paraventricular nucleus. The nucleus accumbens’ role in this circuitry is hypothesized to be a mediator of active avoidance behaviors in face of acute threats^29,42,43^; here, it was derived from the default FreeSurfer parcellation^44^. The hypothalamic nuclei, and particularly its lateral nuclei, are consistently implicated in regulating physiological responses to threat^29,45^; here, it was defined by the lateral hypothalamus ROI from the hypothalamus segmentation^39^. Finally, the periaqueductal gray (PAG) has been shown to generate both passive and active avoidance behaviors and is considered an effector of the defensive response circuit ^46,47^. The PAG ROI was derived from an expert segmentation of brainstem and midbrain nuclei^48^; unlike all other ROIs, which were acquired separately for each hemisphere, the PAG is a single ROI.

In addition to these subcortical structures, we defined several cortical ROIs that have been consistently implicated in defensive responding and threat-anticipatory states. These ROIs were defined using a widely-used, publicly-available parcellation of the cortex derived from resting-state functional connectivity data^49^. This parcellation integrates resting-state data using local gradient and global similarity approaches, yielding parcels that are homogenous in function (as opposed to anatomy-based segmentations). We used the 500-parcel version (v0.14.3). From this parcellation, we selected parcels that correspond or contain our regions of interest (instead of defining a sphere of arbitrary radius which may include heterogeneous portions of multiple regions). The ventromedial prefrontal cortex (equivalent to infralimbic cortex in rodents) is ascribed a major role in fear and defensive responses and their regulation, for example, by contributing to the selection of defense behaviors^29,50^ or by down/up-regulating physiological responses to potential threat via extensive interactions with amygdala, hippocampus, and PAG^51-53^. Recent work further links the posterior vmPFC more specifically with assessment of threat (while the anterior vmPFC is associated with safety assessment)^54^; accordingly, this region was selected here. Left and right vmPFC were defined by the 17Networks_LH_DefaultA_PFCm_2 parcel and the 17Networks_RH_LimbicB_OFC_6 parcels, respectively. Dorsal anterior cingulate cortex (dACC) and dorsomedial PFC (dmPFC) are two regions often implicated in the maintenance of fear responses in humans^38,47,52,55,56^ and the selection and expression of defensive behaviors in animals^50,52^. Here, dACC was defined by the 17Networks_LH_SalVentAttnB_PFCmp_1, 17Networks_LH_SalVentAttnB_PFCmp_3, 17Networks_RH_SalVentAttnB_PFCmp_2, and 17Networks_RH_SalVentAttnB_Cinga_1 parcels. dmPFC was defined by the 17Networks_LH_SalVentAttnA_FrMed_1, 17Networks_LH_SalVentAttnA_FrMed_2, 17Networks_LH_SalVentAttnA_FrMed_3, and 17Networks_RH_SalVentAttnA_FrMed_2 parcels. Anterior insula is hypothesized to contribute to aversive states and defensive behaviors, and consistently emerges in work on threat anticipation^56-60^; it was defined here with the 17Networks_LH_SalVentAttnB_Ins_2 and 17Networks_RH_SalVentAttnB_Ins_2 parcels. Finally, data in humans indicates that dorsolateral prefrontal cortex (dlPFC) is engaged during threat anticipation^56,61^. Given our prior work linking anticipatory physiological response and dlPFC thickness^1,62^, we defined dlPFC using the 17Networks_LH_DefaultA_PFCd_2, 17Networks_LH_DefaultA_PFCd_3, 17Networks_RH_DefaultA_PFCd_2 and 17Networks_RH_DefaultA_PFCd_3.

**ROI merging**. ROIs were all registered to a 1mm resolution image in a standard MNI152 space (MNI152_T1_1mm_brain.nii.gz, as available in FSL, <https://fsl.fmrib.ox.ac.uk/fsl/fslwiki/>). Overlaps between ROIs (from different segmentations) were removed, and all ROIs were then merged into a single 3D image that served as the atlas in analyses. See <https://github.com/rany-abend/atlas> for complete details.

**Results**

**Physiological response to safety and threat cues**

Fig. S2 depicts change in anticipatory skin conductance level from first bin to last bin in the anticipation window, to safety and threat cues.

**
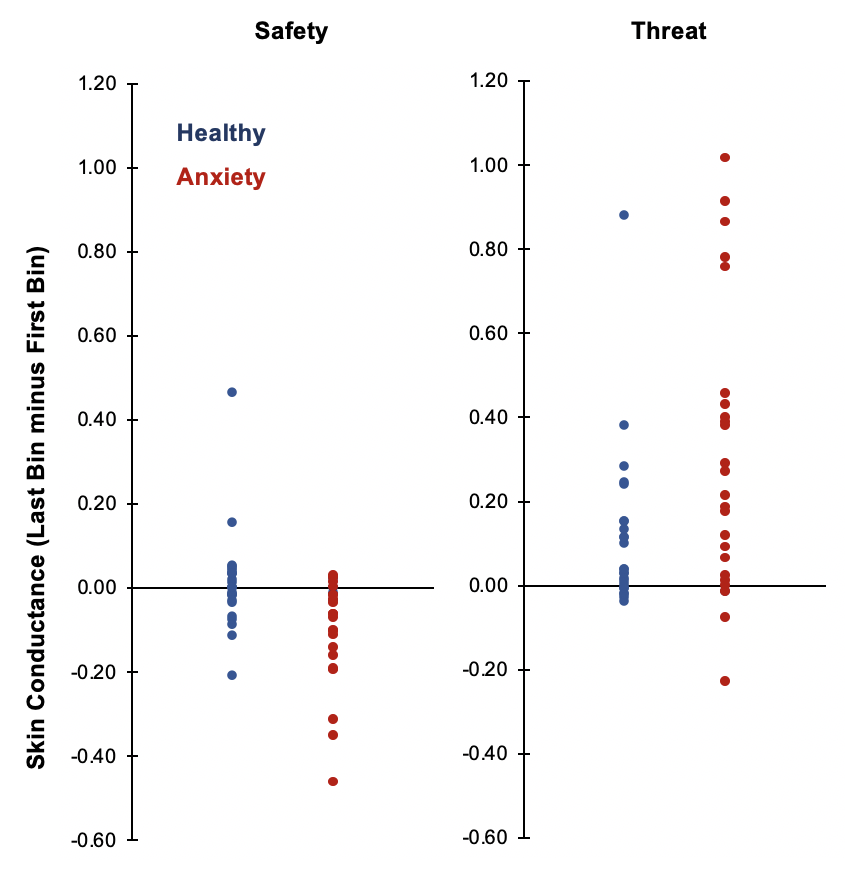
**

**Figure S2.** Change in anticipatory skin conductance level from first bin to last bin, to safety (left) and threat (right) cues for the healthy control (blue) and anxiety (red) groups. Each dot represents data for one participant.

Fig. S3 depicts the Sex×Cue interaction noted on overall physiological response (irrespective of outcome imminence), *F*(1,46)=4.42, *p*=0.041, $\eta_{p}^{2}$=0.09. Decomposition of this interaction by sex indicated that males showed a greater difference in overall response to threat vs. safety cues, *F*(1,15)=23.59, *p*<0.001, $\eta_{p}^{2}$=0.61, relative to females, *F*(1,31)=11.01, *p*=0.002, $\eta_{p}^{2}$=0.26. Decomposition by cue type indicated that females and males did not differ in response to safety cues, *F*(1,46)=0.38, *p*=0.541, $\eta_{p}^{2}$=0.01; males showed a non-significant trend towards higher response to threat cues relative to females, *F*(1,31)=2.85, *p*=0.098, $\eta_{p}^{2}$=0.01.

**
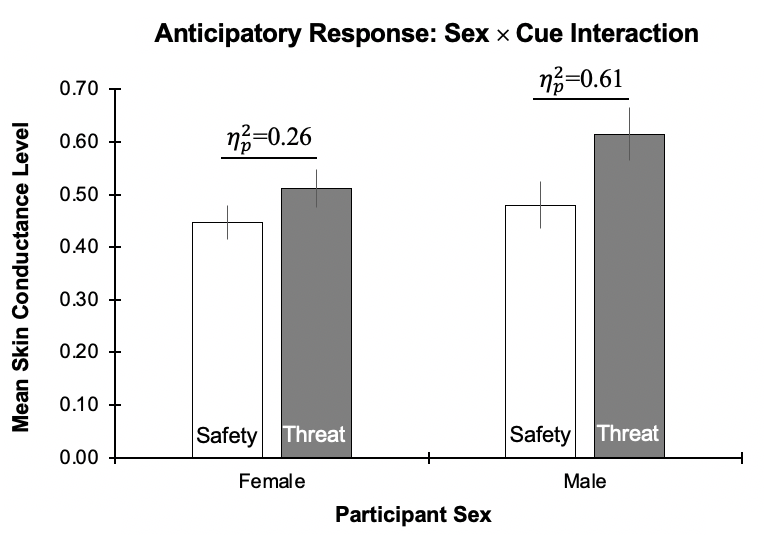
**

**Figure S3.** Average anticipatory skin conductance level (irrespective of outcome imminence) by sex (females, males) and cue type (safety, threat). This interaction is driven by a greater safety vs. threat difference in males relative to females.

***Pre-encounter* and thermal stimulation**

For completeness, we also report on the Cue×Group effect during the period immediately before the cue-based anticipation window, and during thermal stimulation. The pre-cue period, which may correspond to a short *pre-encounter* phase, was defined as a 3s bin prior to cue onset; response was tested with the Cue×Group effect. Analysis indicated a modestly greater physiological response by the anxiety group, *F*(1,48)=5.03, *p*=0.030, $\eta_{p}^{2}$=0.10 (Fig. 2A). No other effects were observed. The correlation between continuous anxiety severity (SCARED scores) and average *pre-encounter* response magnitude (across cues) was positive but not significant, *r*(48)=0.257, *p*=0.071. The primary interaction effect reported above (Cue×Imminence×Group) remained significant even when controlling for *pre-encounter* response magnitude, *F*(1,141)=4.89, *p*=0.003, $\eta_{p}^{2}$=0.09.

Response to the thermal stimulation itself was analyzed similarly during a 4s bin following heat onset. A similar analysis has been reported in our prior work^1^. This analysis indicated a greater response to the highly-painful heat relative to the non-painful heat, as expected, *F*(1,48)=14.94, *p*<0.001, $\eta_{p}^{2}$=0.24. Further, we observed a greater response by the anxiety group (main effect), *F*(1,48)=7.95, *p*=0.007, $\eta_{p}^{2}$=0.14, although this effect was completely abolished once anticipatory response magnitude (bin 4) was controlled for, *p*>0.99. No Cue×Group effect was observed. Similarly, the correlation between continuous anxiety severity and average response to low- and high-pain heat was positive, *r_s_*(48)≥0.359, *p_s_*≤0.011, but became non-significant once anticipatory response was controlled for, |*r_s_*(47)|≤0.018, *p_s_*≥0.900.

**Phasic physiological response**

As an exploratory analysis, we also analyzed the task data using the mean phasic driver instead of the current method. The primary interaction effect of interest, Cue×Imminence×Group, remained significant, although the effect was somewhat weaker than the one currently reported, *F*(3,144)=4.85, *p=*0.003, $\eta_{p}^{2}$=0.09. This suggests that imminence-dependent anxiety effects may be better captured when the overall skin conductance signal is considered, rather than when strictly phasic SCR components are considered. Thus, effects of interested manifest not just through phasic components of the skin conductance signal.

**Intrinsic functional connectivity correlates of physiological response to anticipated threat**

The main text reports on subnetworks within the defensive response network showing significant associations between connectivity and threat-specific increase in anticipatory physiological response, at a predefined threshold of *p*_FDR_<0.05. Fig. S4 depicts connectivity-response correlations at a threshold of *p*<0.05 (uncorrected) to characterize this network more broadly as it relates to anticipatory response. A number of functional connections observed in animal and human studies on fear are replicated in this figure, such as BNST-vmPFC, dmPFC-anterior insula, and vmPFC- and dlPFC-thalamus^38,63,64^.

**
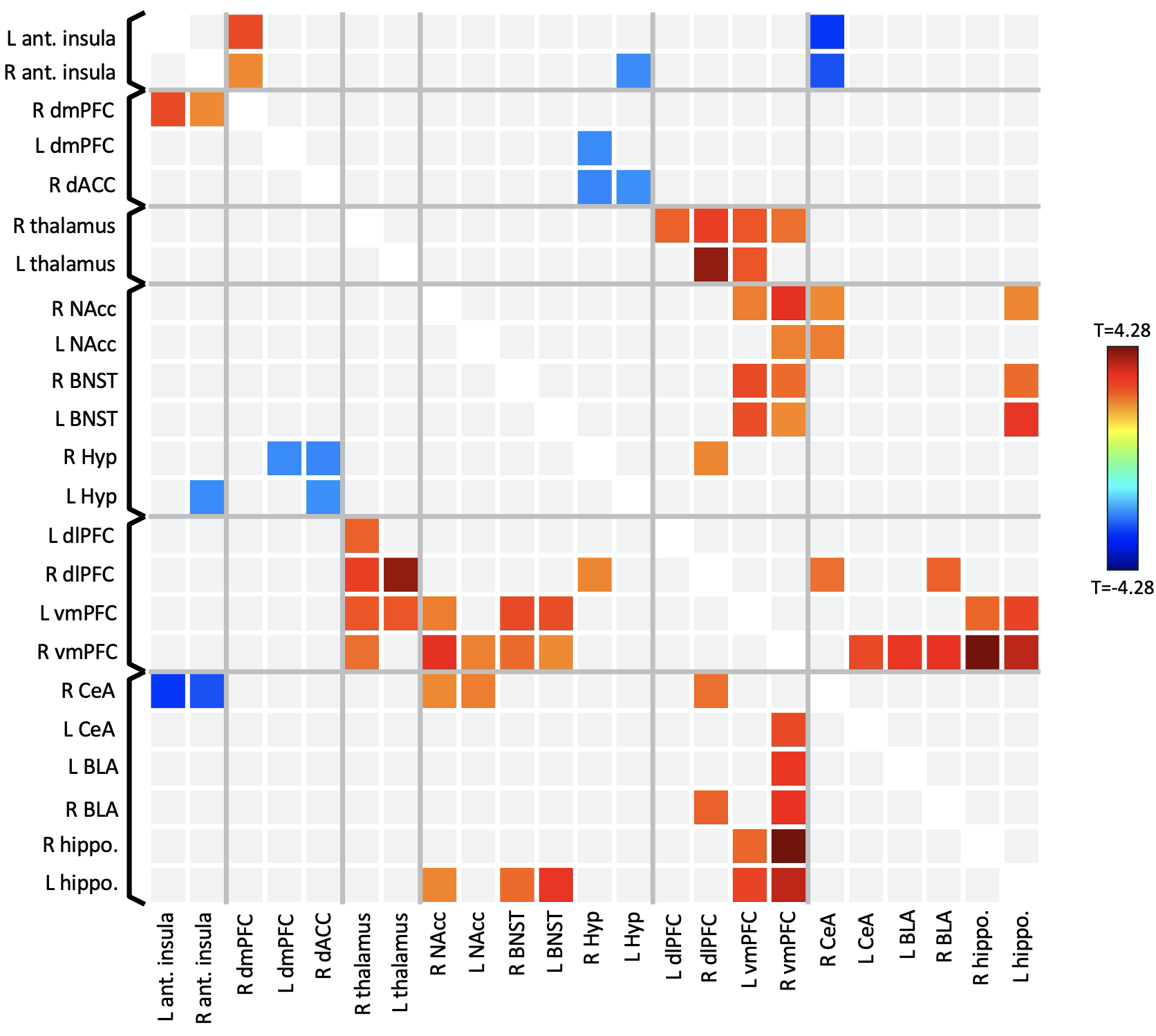
**

**Figure S4.** Correlation magnitudes between network edges and anticipatory physiological responses, thresholded at *p*<0.05 (uncorrected).

*Note*: L=left, R=right, ant. insula=anterior insula, dmPFC=dorsomedial prefrontal cortex, dACC=dorsal anterior cingulate cortex, NAcc=nucleus accumbens, BNST=bed nucleus of stria terminalis, Hyp=hypothalamus, dlPFC=dorsolateral prefrontal cortex, vmPFC=ventromedial prefrontal cortex, CeA=central nucleus of the amygdala, BLA=basolateral amygdala, hippo.=hippocampus.

Fig. S5 depicts edge-wise associations between connectivity and magnitude of initial physiological response (bin 1, averaged across threat and safety cues), at a threshold of *p*<0.05 (uncorrected).


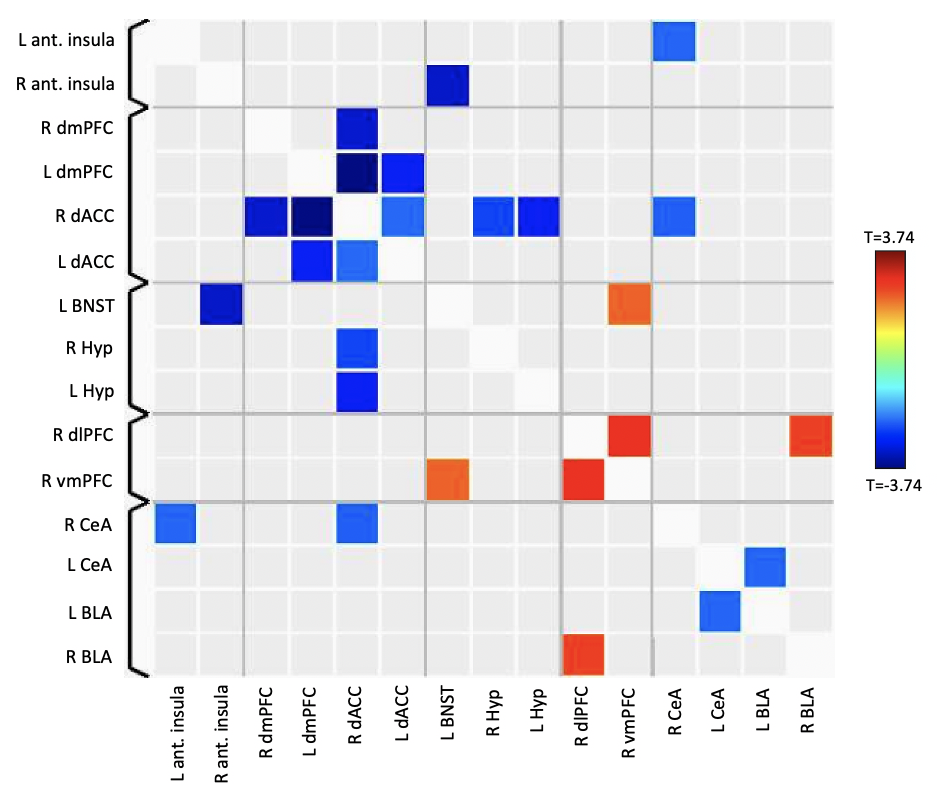


**Figure S5.** Correlation magnitudes between network edges and initial physiological response magnitude (bin 1), thresholded at *p*<0.05 (uncorrected).

*Note*: L=left, R=right, ant. insula=anterior insula, dmPFC=dorsomedial prefrontal cortex, dACC=dorsal anterior cingulate cortex, BNST=bed nucleus of stria terminalis, Hyp=hypothalamus, dlPFC=dorsolateral prefrontal cortex, vmPFC=ventromedial prefrontal cortex, CeA=central nucleus of the amygdala, BLA=basolateral amygdala.


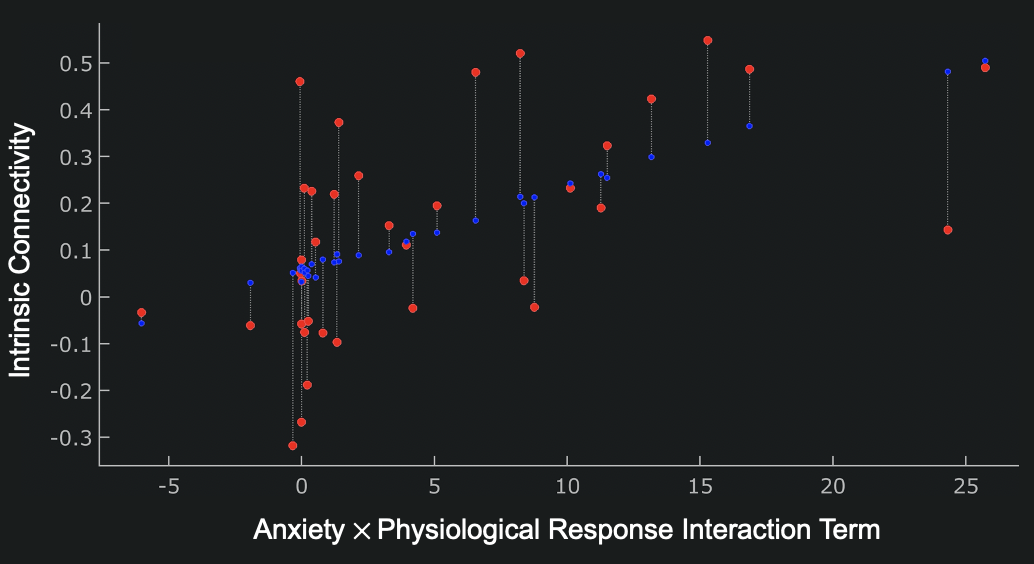


**Figure S6.** Scatterplot depicts association between the interaction of anxiety severity and increase in threat-anticipatory physiological response and the magnitude of intrinsic functional connectivity between right ventromedial prefrontal cortex with bilateral hippocampus and left basolateral amygdala. Connectivity values reflect the average estimates of the three edges. Red dots reflect the observed values; blue dots reflect fitted values on the regression line. Analysis controlled for age.

**References**

1. Abend R, Bajaj M, Harrewijn A, et al. Threat-anticipatory psychophysiological response is enhanced in pediatric anxiety and correlates with prefrontal cortex neuroanatomy. *Journal of Psychiary and Neuroscience.* 2021;46(2).

2. Wechsler D. *Wechsler Abbreviated Scale of Intelligence.* San Antonio, TX (1999): The Psychological Corporation; 1999.

3. Atlas LY, Bolger N, Lindquist MA, Wager TD. Brain mediators of predictive cue effects on perceived pain. *Journal of Neuroscience.* 2010;30(39):12964-12977.

4. Michalska KJ, Feldman JS, Abend R, et al. Anticipatory Effects on Perceived Pain: Associations With Development and Anxiety. *Psychosomatic Medicine.* 2018;80(9):853-860.

5. Hicks CL, von Baeyer CL, Spafford PA, van Korlaar I, Goodenough B. The Faces Pain Scale-Revised: toward a common metric in pediatric pain measurement. *Pain.* 2001;93(2):173-183.

6. *FMRIPrep* [computer program]. 2018.

7. Esteban O, Markiewicz CJ, Blair RW, et al. fMRIPrep: a robust preprocessing pipeline for functional MRI. *Nat Methods.* 2019;16(1):111-+.

8. Gorgolewski KJ, Burns CD, Madison C, et al. Nipype: a flexible, lightweight and extensible neuroimaging data processing framework in python. *Front Neuroinform.* 2011;5:13.

9. *Nipype* [computer program]. 2018.

10. Tustison NJ, Avants BB, Cook PA, et al. N4ITK: Improved N3 Bias Correction. *Ieee Transactions on Medical Imaging.* 2010;29(6):1310-1320.

11. Avants BB, Epstein CL, Grossman M, Gee JC. Symmetric diffeomorphic image registration with cross-correlation: Evaluating automated labeling of elderly and neurodegenerative brain. *Med Image Anal.* 2008;12(1):26-41.

12. Zhang Y, Brady M, Smith S. Segmentation of brain MR images through a hidden Markov random field model and the expectation-maximization algorithm. *IEEE Transactions on Medical Imaging.* 2001;20(1):45–57.

13. Dale AM, Fischl B, Sereno MI. Cortical surface-based analysis. I. Segmentation and surface reconstruction. *Neuroimage.* 1999;9(2):179-194.

14. Klein A, Ghosh SS, Bao FS, et al. Mindboggling morphometry of human brains. *PLOS Computational Biology.* 2017;13(2):e1005350.

15. Fonov V, Evans AC, Botteron K, et al. Unbiased average age-appropriate atlases for pediatric studies. *Neuroimage.* 2011;54(1):313-327.

16. Evans AC, Janke AL, Collins DL, Baillet S. Brain templates and atlases. *Neuroimage.* 2012;62(2):911-922.

17. Cox RW, Hyde JS. Software tools for analysis and visualization of fMRI data. *NMR in Biomedicine.* 1997;10(4-5):171-178.

18. Greve DN, Fischl B. Accurate and robust brain image alignment using boundary-based registration. *Neuroimage.* 2009;48(1):63-72.

19. Jenkinson M, Bannister P, Brady M, Smith S. Improved optimization for the robust and accurate linear registration and motion correction of brain images. *Neuroimage.* 2002;17(2):825-841.

20. Posse S, Wiese S, Gembris D, et al. Enhancement of BOLD-contrast sensitivity by single-shot multi-echo functional MR imaging. *Magn Reson Med.* 1999;42(1):87-97.

21. Pruim RHR, Mennes M, van Rooij D, Llera A, Buitelaar JK, Beckmann CF. ICA-AROMA: A robust ICA-based strategy for removing motion artifacts from fMRI data. *Neuroimage.* 2015;112:267-277.

22. Power JD, Mitra A, Laumann TO, Snyder AZ, Schlaggar BL, Petersen SE. Methods to detect, characterize, and remove motion artifact in resting state fMRI. *Neuroimage.* 2014;84:320-341.

23. Behzadi Y, Restom K, Liau J, Liu TT. A component based noise correction method (CompCor) for BOLD and perfusion based fMRI. *Neuroimage.* 2007;37(1):90-101.

24. Satterthwaite TD, Elliott MA, Gerraty RT, et al. An improved framework for confound regression and filtering for control of motion artifact in the preprocessing of resting-state functional connectivity data. *Neuroimage.* 2013;64:240-256.

25. Lanczos C. Evaluation of Noisy Data. *Journal of the Society for Industrial and Applied Mathematics Series B Numerical Analysis* 1964;1(1):76-85.

26. Abraham A, Pedregosa F, Eickenberg M, et al. Machine learning for neuroimaging with scikit-learn. *Frontiers in Neuroinformatics.* 2014;8:14.

27. Fanselow MS, Dong HW. Are the Dorsal and Ventral Hippocampus Functionally Distinct Structures? *Neuron.* 2010;65(1):7-19.

28. Kirouac GJ. The Paraventricular Nucleus of the Thalamus as an Integrating and Relay Node in the Brain Anxiety Network. *Frontiers in Behavioral Neuroscience.* 2021;15.

29. Mobbs D, Headley DB, Ding WL, Dayan P. Space, Time, and Fear: Survival Computations along Defensive Circuits. *Trends in Cognitive Sciences.* 2020;24(3):228-241.

30. LeDoux J. Emotion circuits in the brain. *Annual Review of Neuroscience.* 2000;23:155-184.

31. Davis P, Reijmers LG. The dynamic nature of fear engrams in the basolateral amygdala. *Brain Research Bulletin.* 2018;141:44-49.

32. Saygin ZM, Kliemann D, Iglesias JE, et al. High-resolution magnetic resonance imaging reveals nuclei of the human amygdala: manual segmentation to automatic atlas. *Neuroimage.* 2017;155:370-382.

33. Fadok JP, Markovic M, Tovote P, Luthi A. New perspectives on central amygdala function. *Current Opinion in Neurobiology.* 2018;49:141-147.

34. Ledoux JE, Iwata J, Cicchetti P, Reis DJ. Different Projections of the Central Amygdaloid Nucleus Mediate Autonomic and Behavioral-Correlates of Conditioned Fear. *Journal of Neuroscience.* 1988;8(7):2517-2529.

35. Iglesias JE, Augustinack JC, Nguyen K, et al. A computational atlas of the hippocampal formation using ex vivo, ultra-high resolution MRI: Application to adaptive segmentation of in vivo MRI. *Neuroimage.* 2015;115:117-137.

36. Odriozola P, Gee DG. Learning About Safety: Conditioned Inhibition as a Novel Approach to Fear Reduction Targeting the Developing Brain. *American Journal of Psychiatry.* 2021;178(2):136-155.

37. Lebow MA, Chen A. Overshadowed by the amygdala: the bed nucleus of the stria terminalis emerges as key to psychiatric disorders. *Molecular Psychiatry.* 2016;21(4):450-463.

38. Robinson OJ, Pike AC, Cornwell B, Grillon C. The translational neural circuitry of anxiety. *J Neurol Neurosur Ps.* 2019;90(12):1353-1360.

39. Neudorfer C, Germann J, Elias GJB, Gramer R, Boutet A, Lozano AM. A high-resolution in vivo magnetic resonance imaging atlas of the human hypothalamic region. *Sci Data.* 2020;7(1).

40. Salay LD, Ishiko N, Huberman AD. A midline thalamic circuit determines reactions to visual threat. *Nature.* 2018;557(7704):183-+.

41. Iglesias JE, Insausti R, Lerma-Usabiaga G, et al. A probabilistic atlas of the human thalamic nuclei combining ex vivo MRI and histology. *Neuroimage.* 2018;183:314-326.

42. Ramirez F, Moscarello JM, LeDoux JE, Sears RM. Active Avoidance Requires a Serial Basal Amygdala to Nucleus Accumbens Shell Circuit. *Journal of Neuroscience.* 2015;35(8):3470-3477.

43. Mobbs D. The ethological deconstruction of fear(s). *Curr Opin Behav Sci.* 2018;24:32-37.

44. Fischl B, Salat DH, Busa E, et al. Whole brain segmentation: automated labeling of neuroanatomical structures in the human brain. *Neuron.* 2002;33(3):341-355.

45. Adolphs R. The Biology of Fear. *Current Biology.* 2013;23(2):R79-R93.

46. Lefler Y, Campagner D, Branco T. The role of the periaqueductal gray in escape behavior. *Current Opinion in Neurobiology.* 2020;60:115-121.

47. Mobbs D, Petrovic P, Marchant JL, et al. When fear is near: Threat imminence elicits prefrontal-periaqueductal gray shifts in humans. *Science.* 2007;317(5841):1079-1083.

48. Edlow BL, Takahashi E, Wu ON, et al. Neuroanatomic Connectivity of the Human Ascending Arousal System Critical to Consciousness and Its Disorders. *J Neuropath Exp Neur.* 2012;71(6):531-546.

49. Schaefer A, Kong R, Gordon EM, et al. Local-Global Parcellation of the Human Cerebral Cortex from Intrinsic Functional Connectivity MRI. *Cerebral Cortex.* 2018;28(9):3095-3114.

50. Halladay LR, Blair HT. Distinct ensembles of medial prefrontal cortex neurons are activated by threatening stimuli that elicit excitation vs. inhibition of movement. *J Neurophysiol.* 2015;114(2):793-807.

51. Zhang S, Hu S, Chao HH, et al. Ventromedial prefrontal cortex and the regulation of physiological arousal. *Social Cognitive and Affective Neuroscience.* 2014;9(7):900-908.

52. Milad MR, Quirk GJ. Fear Extinction as a Model for Translational Neuroscience: Ten Years of Progress. *Annual Review of Psychology.* 2012;63:129-151.

53. Myers-Schulz B, Koenigs M. Functional anatomy of ventromedial prefrontal cortex: implications for mood and anxiety disorders. *Molecular Psychiatry.* 2012;17(2):132-141.

54. Tashjian SM, Zbozinek TD, Mobbs D. A Decision Architecture for Safety Computations. *Trends in Cognitive Sciences.* 2021;25(5):342-354.

55. Fullana MA, Harrison BJ, Soriano-Mas C, et al. Neural signatures of human fear conditioning: an updated and extended meta-analysis of fMRI studies. *Molecular Psychiatry.* 2016;21(4):500-508.

56. Grupe DW, Nitschke JB. Uncertainty and anticipation in anxiety: an integrated neurobiological and psychological perspective. *Nature Reviews Neuroscience.* 2013;14(7):488-501.

57. Holzschneider K, Mulert C. Neuroimaging in anxiety disorders. *Dialogues Clin Neurosci.* 2011;13(4):453-461.

58. Rogers-Carter MM, Varela JA, Gribbons KB, et al. Insular cortex mediates approach and avoidance responses to social affective stimuli. *Nature Neuroscience.* 2018;21(3):404-+.

59. Carlson JM, Greenberg T, Rubin D, Mujica-Parodi LR. Feeling anxious: anticipatory amygdalo-insular response predicts the feeling of anxious anticipation. *Social Cognitive and Affective Neuroscience.* 2011;6(1):74-81.

60. Drabant EM, Kuo JR, Ramel W, et al. Experiential, autonomic, and neural responses during threat anticipation vary as a function of threat intensity and neuroticism. *Neuroimage.* 2011;55(1):401-410.

61. Aupperle RL, Allard CB, Grimes EM, et al. Dorsolateral prefrontal cortex activation during emotional anticipation and neuropsychological performance in posttraumatic stress disorder. *Archives of General Psychiatry.* 2012;69(4):360-371.

62. Abend R, Gold AL, Britton JC, et al. Anticipatory Threat Responding: Relationships with Anxiety, Development, and Brain Structure. *Biological Psychiatry.* 2020;87(10):916-925.

63. Ganella DE, Drummond KD, Ganella EP, Whittle S, Kim JH. Extinction of Conditioned Fear in Adolescents and Adults: A Human fMRI Study. *Frontiers in Human Neuroscience.* 2017;11:647.

64. Tao Y, Cai CY, Xian JY, et al. Projections from Infralimbic Cortex to Paraventricular Thalamus Mediate Fear Extinction Retrieval. *Neuroscience Bulletin.* 2021;37(2):229-241.
